# Spatial meta-analysis of the occurrence and distribution of tsetse-transmitted animal trypanosomiasis in Cameroon over the last 30 years

**DOI:** 10.1017/S0950268822000772

**Published:** 2022-04-27

**Authors:** Silas Lendzele Sevidzem, Aubin Armel Koumba, Jacques François Mavoungou, Peter Andrew Windsor

**Affiliations:** 1 Programme Onchocercoses Field Station Laboratory, Ngaoundéré, Cameroon; 2 Organisation Pour la Production Laitière et d'Embouche Bovine (PLEB), Adamawa, Cameroon; 3 Laboratoire d'Ecologie Vectorielle, Département de Biologie et Ecologie Animale, Institut de Recherche en Ecologie Tropicale, Libreville, Gabon; 4 Université Internationale de Libreville, Libreville, Gabon; 5 Université des Sciences et Techniques, Franceville, Gabon; 6 The University of Sydney, Camden, NSW, Australia

**Keywords:** Abundance, African animal trypanosomiasis, Cameroon, distribution, prevalence, tsetse

## Abstract

In Cameroon, >90% of cattle are considered exposed to African animal trypanosomiasis (AAT) infection, with the presence of tsetse rendering cattle husbandry as a very difficult proposition. A systematic review of data on AAT and tsetse from 1990 to 2021 was conducted to develop a national atlas. The review identified 74 relevant scientific documents, with three pathogenic *Trypanosoma* species (*Trypanosoma vivax*, *T. congolense* and *T. brucei* s.l.) most frequently identified as causing AAT. *Trypanosoma grayi*, *T. theileri*, *T. simiae* and the human African trypanosomiasis causative agent *T. brucei gambiense* were also identified in a wide range of hosts. The tsetse fly fauna of Cameroon comprises nine species, with *Glossina palpalis palpalis* and *G. fuscipes fuscipes* the most widely distributed following their identification in seven and five of the 10 regions, respectively. Two species, *Glossina nigrofusca* and *G. pallicera pallicera* appeared to be rare and were restricted to both forest and protected areas. The presence of AAT is associated with the presence of tsetse in the livestock–human–wildlife interface of Cameroon. AAT occurs beyond the tsetse belts of the country where mechanical vectors are abundant. This study provides AAT and tsetse maps to support ongoing interventions in Cameroon.

## Introduction

African animal trypanosomiasis (AAT) also recognised as ‘nagana’, is a family of blood dwelling parasitic diseases caused by the members of the genus *Trypanosoma* [1]. In animals especially cattle, AAT is frequently caused by *Trypanosoma vivax*, *T. congolense* and *T. brucei* s.l. [2]. However, other *Trypanosoma* spp. have been diagnosed in animals, including *Trypanosoma simiae* (in pigs, monkeys, etc.), *T. theileri* (in cattle, sheep, goats), *T. grayi* (in crocodiles) and *T. evansi* (in camels, horses) [3, 4]. Studies from Cameroon and elsewhere in Africa have shown that AAT-causing trypanosomes, including *T. vivax*, *T. congolense* and *T. brucei* s.l., have been isolated from wild animals [5, 6]. AAT is one of the most common and important veterinary diseases in sub-Saharan Africa (SSA), causing mortalities of several thousands of heads of cattle annually and morbidities that diminish animal productivity, through losses of meat, milk, draught power, fertility, manure and abortion, leading to deaths if not treated early [7].

In Cameroon, >90% of the estimated 6 million cattle are predisposed to trypanosomiasis [8]. The first cases of AAT in Cameroon were declared in 1943 in the Adamawa region [9]. It is known that tsetse-transmitted AAT (TTAAT) extends in SSA over 10 million km^2^. Apart from its cyclical or biological transmission by tsetse flies, AAT can be mechanically transmitted through the infective bites of some dipterids, including *Stomoxys* and tabanids [10–13]. In the tsetse fly-free belts of tropical Africa, AAT exists in the apparent absence of tsetse flies, with transmission in these areas reported to be due to other dipterous flies and possibly hard-ticks carrying the parasite [14–20]. Cameroon is not exempt from non-tsetse-transmitted AAT (NTTAAT) as cattle in tsetse-free areas of Ngaoundere and the Far North region have been reported with trypanosome infections in the apparent absence of tsetse flies [11, 12, 21]. Furthermore, 4.02% of cattle from different ranches of tsetse-free Ngaoundere have been diagnosed with *T. congolense* and *T. vivax* [22]. Tabanids, especially *Chrysops longicornis*, *Haematopota decora* and *Tabanus taeniola* caught from tsetse-free Ngaoundere, have been confirmed by molecular studies to harbour trypanosomes [11]. These reports indicate that biting flies could still sustain the transmission of AAT in the apparent absence of tsetse flies.

For TTAAT, tsetse flies are important for transmission and epidemiology of the disease [1, 23]. Tsetse flies belong to the genus *Glossina* and are regrouped under three subgenera: Austenina (fusca group), Nemorhina (palpalis group) and Morsitans (morsitans group). Tsetse flies occur exclusively in SSA over an area of approximately 10 million km^2^. They are known to comprise 34 species and subspecies [24]. According to the technical report of the Special Mission for Tsetse fly Eradication (MSEG) of 2005 [11], 12 species of tsetse flies occurs in Cameroon including: (1) Fusca group including *Glossina fusca congolensis*, *G. tabaniformis*, *G. nigrofusca*, *G. fuscipleuris*, *G. haningtoni*; (2) Palpalis group including *G. palpalis palpalis*, *G. fuscipes fuscipes*, *G. tachinoides*, *G. caliginea*, *G. pallicera pallicera* and (3) Morsitans group including *G. morsitans submorsitans* and *G. longipalpalis*.

The occurrence of tsetse flies in SSA has made livestock husbandry very difficult, with historical and current regional and international efforts to establish significant tsetse-free areas and minimise re-infestation. In the Adamawa region of Cameroon, three *Glossina* species (*G. morsitans submorsitans*, *G. fuscipes fuscipes* and *G. tachinoides*) are commonly reported [25]. Eradication efforts between the 1960s and 1990s in the Adamawa plateau, led to a tsetse-free area of up to 3 200 000 hectares of rangeland, although 20 years later, such areas were confronted with re-infestation [9, 26].

Current entomological reports from various regions indicate the widespread occurrence of tsetse flies, including Adamawa [27–31], North [32, 33], East [34, 35], North West [36], South West [37, 38], Centre [39], Littoral [40], West [41] and South [42, 43]. However, reports from the Far North region indicate the apparent absence of this fly-group [44]. Examination of this existing data on the distribution of tsetse flies in Cameroon suggests that updated distribution and density maps are required for improving the identification of areas for risk-based AAT control (Control measures that are selected based on their effectiveness at reducing the probability and impact of AAT spread).

In Cameroon, cattle rearing is a major activity of individuals living in rural poor areas of the Far North, North, Adamawa, East and North West. In the major cattle-rearing regions, particularly the Adamawa plateau, cattle husbandry is greatly compromised by bovine trypanosomiasis [45, 46]. Because AAT is an economically devastating disease and a major threat to the livestock health and production in the country, the Government of Cameroon embarked on eradication activities between the 1960s and 1970s in the major cattle-rearing areas of Adamawa, Far North, North and East [9]. These control efforts were directed towards mass treatment of cattle with trypanocides and aerial insecticide spraying of pasture areas. Despite the enormous efforts to free the rangelands from tsetse and the diseases they transmit, the re-invasion of previously freed areas still occurred [9]. In 2005, the first report on trypanocidal drug resistance was made in the Adamawa region of Cameroon [9, 47]. More than 10 years later, trypanocidal resistance of *T. congolense* isolates in tsetse flies and ruminants (cattle and sheep) from Yoko in the Centre region of Cameroon was reported [39, 48].

From 1974 to until now, AAT control activities have been conducted by the MSEG of the Ministry of Livestock, Fisheries and Animal Industries (MINEPIA), focused mainly on major cattle breeding areas. The interventions in the Adamawa plateau led to the freeing of about 8 000 000 ha of pasture land from tsetse flies [49].

The risk-based control strategy is considered an important approach for cost-effective disease mitigation. This idea triggered the creation of the progressive control pathway (PCP), a step-wise methodology originally developed for foot-and-mouth disease [50], and now used for other diseases including AAT [50]. Applying this AAT-PCP in the Cameroonian context by the MSEG, it was estimated that the country is in step 1 (characterised by capacity development, understanding risk and impact, selection of priority intervention areas and intervention strategies), with the Adamawa in step 2 (characterised by the integrated management of AAT (community/farm-based, supervised by veterinary services), sustainable and economically profitable reduction in AAT burden), whilst the Far North is in step 3 (elimination of AAT transmission) [49]. However, there is a need for updated data on the tsetse and trypanosomiasis distribution, particularly to inform risk-based control activities for tsetse flies and AAT in Cameroon. The main objective of this study is to develop a national atlas for tsetse and AAT to facilitate interventions in Cameroon.

## Materials and methods

### Study country

**Fig. 1.**
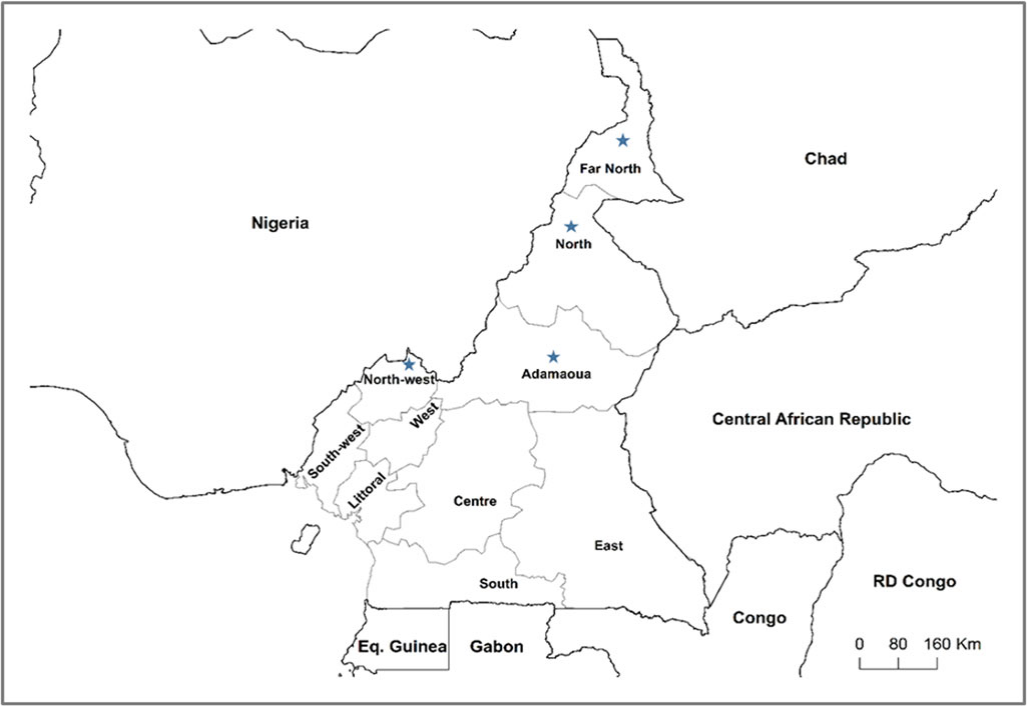
Map of Cameroon and neighbouring countries of the central African subregion. The blue stars (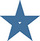) indicate the major cattle-rearing regions.

This study was conducted in Cameroon, a country in the northeastern part of the Gulf of Guinea with a total surface area of 475 000 km^2^, located between longitude 9°0′0″–16°0′0″ east and latitude 2°0′0″–13°0′0″ north. Cameroon is part of the Central African states, sharing a boundary to the south with Equatorial Guinea, Gabon and Congo, to the west with Nigeria, to the east with the Central African Republic and Chad (Fig. 1). The country comprises 10 regions with five agro-ecological zones (Sudan savannah, Guinea savannah, highland plateau, rainforest and Mosaic forest). Five (Adamawa, North West, Far North, North and East) of the 10 regions are major cattle breeding areas with the Adamawa region known as the supplier of meat to the central African subregion [51]. The population of Cameroon in 2020 was estimated at about 26 000 000 inhabitants (National Institute for Statistics; NIS). In a report published in 2015, the livestock population included 31 million poultry, 6 million cattle, 7 million small ruminants, 1 million pigs, 150 000 donkeys and 15 000 horses [52].

### Article search and creation of database

**Fig. 2.**
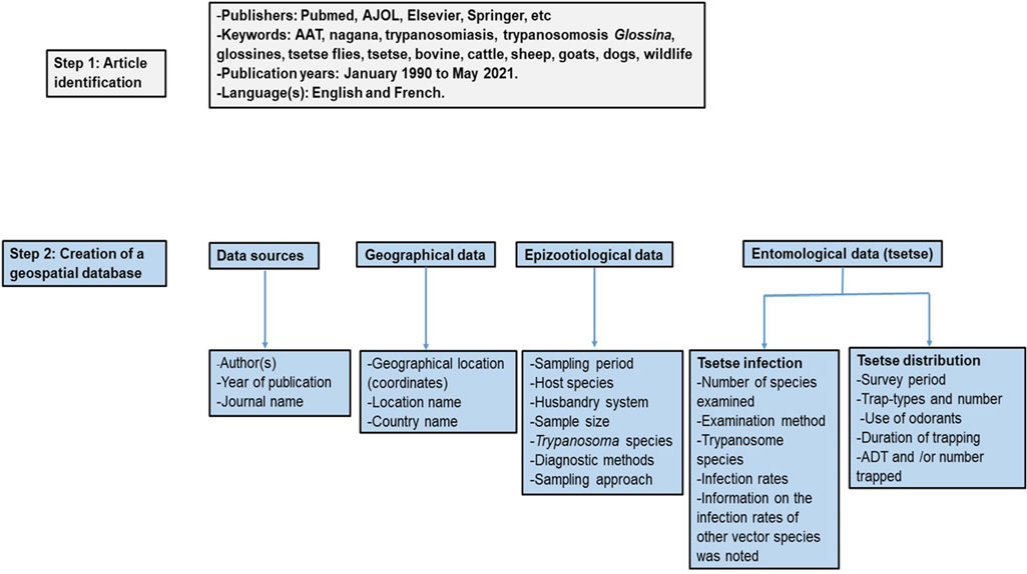
Workflow on the steps for information search and creation of AAT and tsetse flies database.

The steps established by the FAO for the development of a continental atlas of tsetse and AAT have previously been described [53–55] with the present study using these methods to develop AAT and tsetse distribution maps for Cameroon. The present study used this method proposed by the FAO because the idea is to have a harmonised protocol for the different African countries in order to facilitate the development of a continental atlas for tsetse and AAT. The methodology is summarised (Fig. 2). An online search via Google, Google Scholar, Academia and ResearchGate to get documents on tsetse and AAT in Cameroon was conducted using the following keywords: AAT, nagana, trypanosomiasis, trypanosomosis Glossina, glossines, tsetse flies, tsetse, bovine trypanosomiasis, cattle trypanosomiasis, sheep trypanosomes, trypanosomes in goats trypanosomes, trypanosomes in dogs, trypanosomiasis in wildlife. Additionally, the annual reports of the MSEG (the only division involved in the fight against tsetse flies and trypanosomiasis in Cameroon) were considered. The dissertations defended on AAT and tsetse in Cameroonian universities (University of Dschang, University of Ngaoundere, University of Yaounde 1). The following inclusion criteria were considered for the selection of documents: (i) only documents available from January 1990 to May 2021 were considered and (ii) documents with both epidemiological and entomological data were considered as one in order to avoid duplications. The following criteria were considered for the exclusion of documents: (i) review papers were excluded, (ii) papers and dissertations reporting on experimental infections and human African trypanosomiasis (HAT) were not included in the database, (iii) entomological data on mechanical vectors such as tabanids and *Stomoxys* were noted but not included in the database and (iv) the infection rates (IRs) of mechanical vectors such as tabanids and *Stomoxys* were noted but not included in the database.

### Creation of the atlas

The extracted data from different sources into the atlas databases required a systematic process of verification and harmonisation. Geographical coordinates were standardised as latitude and longitude and their conversion was conducted where required. Data extracted from selected documents were compiled in Access 2013 (Microsoft) databases, then joined to the locality shape files produced from gpx files from GPS handsets in ArcMap™ version 10.1, using Geographic Information System software (Environmental Systems Research Institute, USA).

## Results

Every year, the Government of Cameroon provides financial support to MSEG to fight against tsetse and trypanosomiasis. The aims of MSEG are (i) to free tsetse-infested rangelands, (ii) to treat animals using trypanocides and (iii) to sensitise farmers on how to control tsetse and AAT. For more than half a century of tsetse flies and trypanosomiasis fight in Cameroon, AAT is still problematic in major cattle-rearing regions. To date, no robust nationwide data have been collected to map AAT and tsetse hotspot areas for targeted control. This is an attempt to map reliable and updated information on tsetse and AAT in the different regions of Cameroon to support ongoing interventions.

### Characteristics of the data extracted from scientific documents

Although a total of 151 results were obtained from online searches, only 74 scientific documents met the criteria for sufficiently complete information (meaning articles with main aspects of epizootiology of AAT and/or tsetse) on the occurrence of AAT, tsetse and their trypanosomal infection were identified as suitable for inclusion in the study. In particular, 34 papers provided information on AAT distribution (proportion of the number of infected animals in study sites), 28 on tsetse distribution (tsetse abundance data at trap-points) and 12 on tsetse trypanosomal IRs. The breakdown of papers by publication year is provided (Appendix Table A1), and shows that more than half of the papers were published in the last 10 years. Appendix Table A2 summarises the availability of published surveys and the reported presence of AAT, tsetse flies and tsetse trypanosomal infection for the different regions of Cameroon.

### AAT prevalence findings

**Fig. 3.**
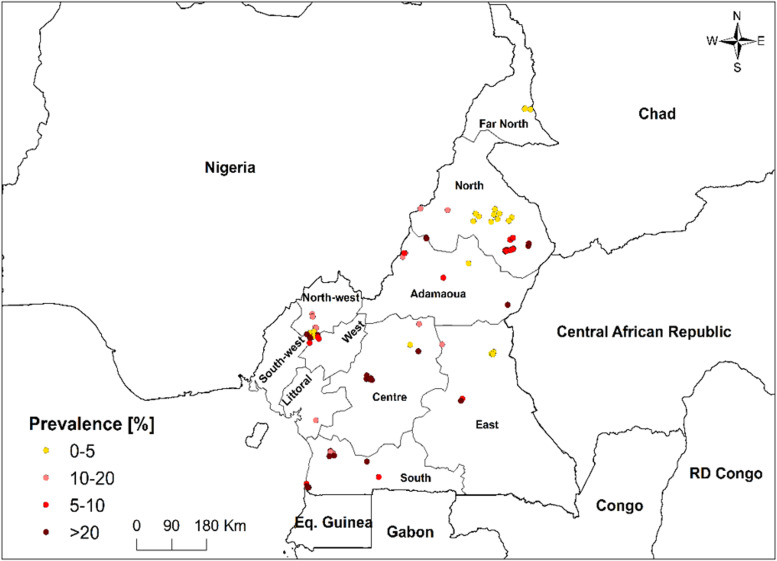
Map of AAT prevalence in buffy coat collected in Cameroon in 1990–2021. The number of studies, number of examined animals and mean AAT prevalences, respectively, per region were: Adamaoua (12; 6558; 28.24%), Centre (2; 848; 25.50%), East (2; 690; 2.82%), Far North (1; 770; 1.46%), North (5; 4391; 21.71%), North West (1; 301; 10.3%), South (4; 3368; 16.77%), South West (7; 2606; 22.76%).

Thirty four studies and 338 survey sites (herds, villages, subdivisions and divisions) were involved in the AAT investigations. In total, 19 532 animals were examined for AAT in eight regions of Cameroon, with an overall infection prevalence of 21% recorded across animal species and study region. The infection prevalence within animal species revealed highest rate in pigs followed by sheep, goats, wildlife, cattle and lastly in dogs. Regarding the infection prevalence rates by study regions, the highest prevalences occurred in the Adamawa and Centre regions. However, lowest prevalence rates occurred in the East and Far North regions (Fig. 3).

### AAT distribution

**Fig. 4.**
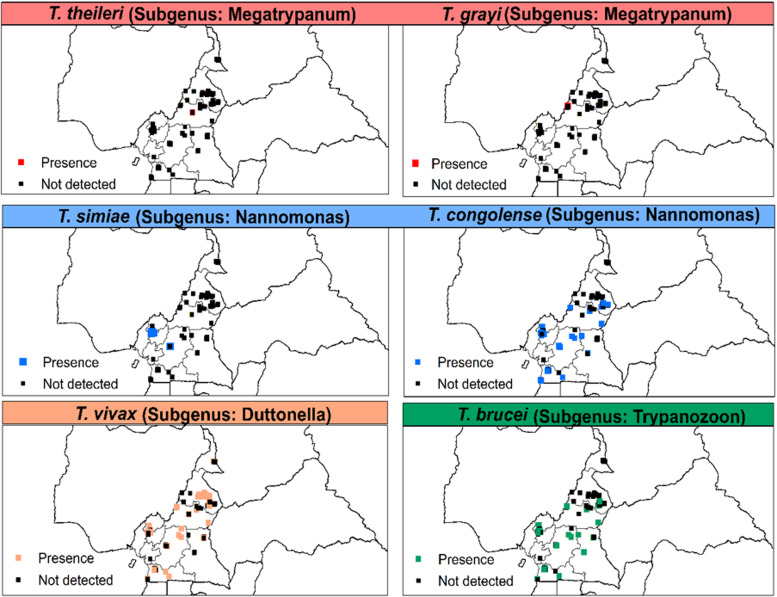
Distribution of *Trypanosoma* spp. of the subgenera *Megatrypanum*, *Nannomonas*, *Duttonella* and *Trypanozoon* based on microscopic and/or molecular methods from 1990 to 2021. *T. theileri* and *T. grayi* were based on polymerase chain reaction (PCR)-based methods only.

AAT was detected in animals of eight out of the 10 regions of Cameroon. *T. congolense* (‘forest’ and ‘savannah’ types), *T. vivax* and *T. brucei* s.l. were most frequent. However, *T. grayi* was only detected in cattle from Kontcha in the Adamawa region. *T. simiae* was identified in animals from the southern part of the country while *T. theileri* was only identified in the northern regions (Adamawa and North) (Fig. 4).

### Tsetse distribution findings

**Fig. 5.**
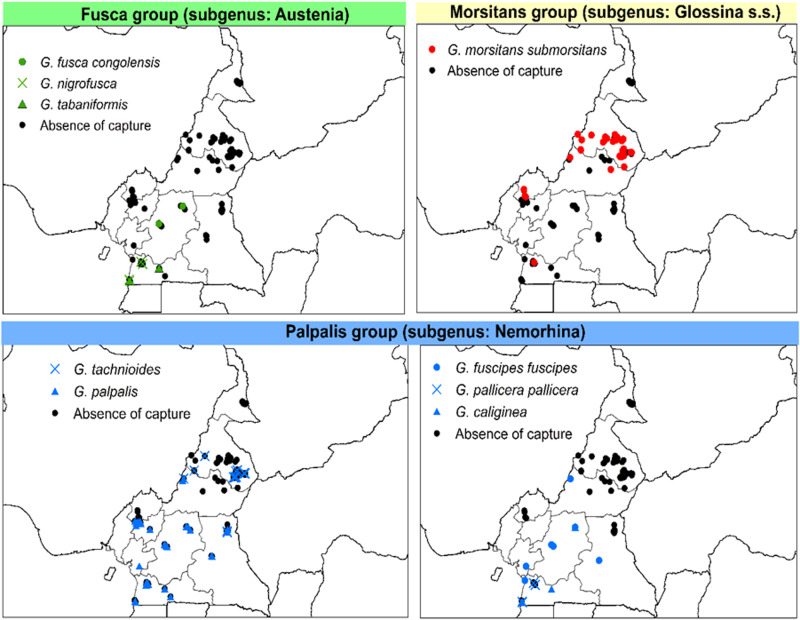
Distribution of *Glossina* spp. of subgenera *Austenina*, *Nemorhina* and *Glossina* based on morphological identification.

For the regional distribution of *Glossina* species, it was found that *G. palpalis palpalis* was the most widely distributed species, followed by *G. fuscipes fuscipes* and *G. fusca congolensis*. However, *G. pallicera pallicera* and *G. nigrofusca* were rare and found only in the southern regions (Fig. 5).

### Tsetse trap apparent density (ADT) and IRs

**Fig. 6.**
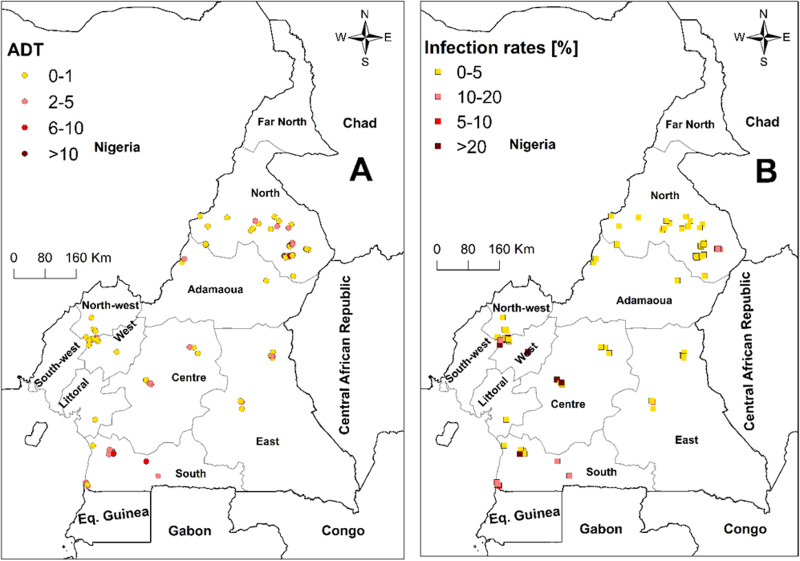
Map showing the ADT of tsetse flies (A) and their trypanosomal IRs (B). All the studies on IRs used PCR methods for diagnosis.

For the tsetse surveys, 28 studies were documented, involving 650 trapping sites in nine regions. The following traps were used: biconical, Vavoua, Nzi, Sevi, pyramidal, modified Vavoua and H-trap. The most commonly encountered traps in the different studies were biconical, Nzi and Vavoua. The entomological data from the different sources indicated that trap types and numbers differed between studies. The total number of tsetse flies caught in these regions was 52 513, with abundance (ADT) of 1.5 flies per trap per day (f/t/d). Based on the ADT within regions, the highest value was recorded in the Littoral and South West. Lowest ADTs were recorded in the West and East regions. No tsetse was reported in the Far North region of Cameroon (Fig. 6A). For the tsetse trypanosomal IRs, the overall average prevalence was 14.3% in six regions of the country. The tsetse IRs with region in the order of magnitude were: North; West; Adamawa; Centre; South and South West (Fig. 6B).

### Occurrence of *Trypanosoma* spp. in tsetse flies

The pathogenic *T. congolense* and *T. brucei* s.l. were detected in tsetse flies from all the six regions. *T. vivax* was reported in tsetse flies from southern Cameroon (South West, Centre and West). *T. grayi* was only detected in tsetse from northern regions (Adamawa and North) (Table 1). A study in the tsetse-free zone of Ngaoundere in the Adamawa plateau identified *T. vivax*, *T. theileri* and *T. evansi* in *C. longicornis*.

**Table 1.** *Trypanosoma* spp. distribution in tsetse flies in the different regions

Region	*Trypanosoma* spp. identified in tsetse flies				
	*T. congolense*	*T. vivax*	*T. brucei* s.l.	*T. grayi*^a^	References
South	+	−	+	−	[42, 43, 56]
Adamawa	+	−	+	+	[30, 33]
South West	+	+	+	−	[57–60]
North	+	−	+	+	[33]
Centre	+	+	+	−	[39, 61]
East	NA	NA	NA	NA	
Littoral	NA	NA	NA	NA	
West	+	+	+	−	[41]
North West	NA	NA	NA	NA	
Far North	NA	NA	NA	NA	

The plus (+) sign in the table indicate the presence of *Trypanosoma* species and minus (−) sign depicts its absence in tsetse in the region. NA indicates that no study has been conducted on tsetse trypanosomal species identification.

a Molecular detection only.

### Occurrence of other haematophagous dipterids

Apart from tsetse flies, biting flies such as tabanids and *Stomoxys* were both highly abundant and diverse, in all the agro-ecological zones of Cameroon. The tabanid fauna encountered in most papers included: (i) in genus *Tabanus*, 11 species, including *T. taeniola*, *T. secedens*, *T. latipes*, *T. par*, *T. argenteus*, *T. gratus*, *T. fasciatus*, *T. rufricrus*, *T. sufis*, *T. biguttatus* and *T. socius*; (ii) in genus *Chrysops*, five species: *C. longicornis*, *C. dimidiata*, *C. silacea*, *C. distinctipennis* and *C. funebris*; (iii) in genus *Haematopota*, three species: *H. decora*, *H. nigripennis* and *H. pluvialis*; (iv) in genus *Ancala*, one species: *Ancala fasciata* and (v) in genus *Atylotus*, one species: *Atylotus agrestis*. Biting Muscidae, genus *Stomoxys*, were also identified and included: *Stomoxys niger niger*, *S. n. bilineatus*, *S. calcitrans*, *S. omega*, *S. sitiens*, *S. xanthomelas*, *S. transvittatus* and *S. inornatus*. Other biting dipterids encountered in livestock settlement areas included nematocerans of the following genera: *Culex*, *Anopheles*, *Culicoides* and *Simulium*. In the Far North region of Cameroon, it was noticed that the above-mentioned biting dipterous insects showed affinity for following hosts: cattle, sheep, goats, dogs, horses, donkeys, poultry and humans in settlement areas. In the Far North, *T. vivax* was detected in cattle in the apparent absence of tsetse flies, although high density of tabanids was noted, with the suggestion that *T. vivax* could be transmitted mechanically via the infective bite of tabanids. This has been confirmed by molecular studies conducted in tsetse-free Ngaoundere in the Adamawa region, where *C. longicornis* was reported to harbour *T. vivax*, *T. theileri* and *T. evansi*. Because of the increasing NTTAAT in some tsetse-free belts in the northern region of Cameroon, the MSEG is preparing to include these biting flies in fly control operations.

### AAT, tsetse and other biting flies monitoring surveys

From the available data, only four papers had information on AAT and vector monitoring. The studies in three of the four papers were conducted in the tsetse-infested areas of the northern regions of Cameroon (Adamawa and North). Only one of the papers presented results on the monitoring of biting insects (genera *Culex*, *Anopheles*, *Culicoides*, *Stomoxys*, *Tabanus*, *Chrysops*, *Haematopota* and *Simulium*) in the tsetse-free zone of Ngaoundere. The study on the AAT monitoring in the Adamawa plateau indicated that peak IR was 44.8% in the control group yet only 10% in the treatment (treatment was conducted using VERIBEN (diminazene aceturate, 7 mg/kg)) group. The study in the North focused on the monitoring of tsetse apparent densities and revealed baseline ADT of 28.9 f/t/d, with an ADT of 18.4 f/t/d after controls were applied. For the monitoring study on biting insects in Ngaoundere, the baseline ADT was 21.8 f/t/d, with an ADT of 5.7 f/t/d after control was applied. Despite these findings, it is clear that available online data on tsetse and AAT monitoring in Cameroon remain scant.

### Mapping AAT presence and tsetse presence data in Cameroon

**Fig. 7.**
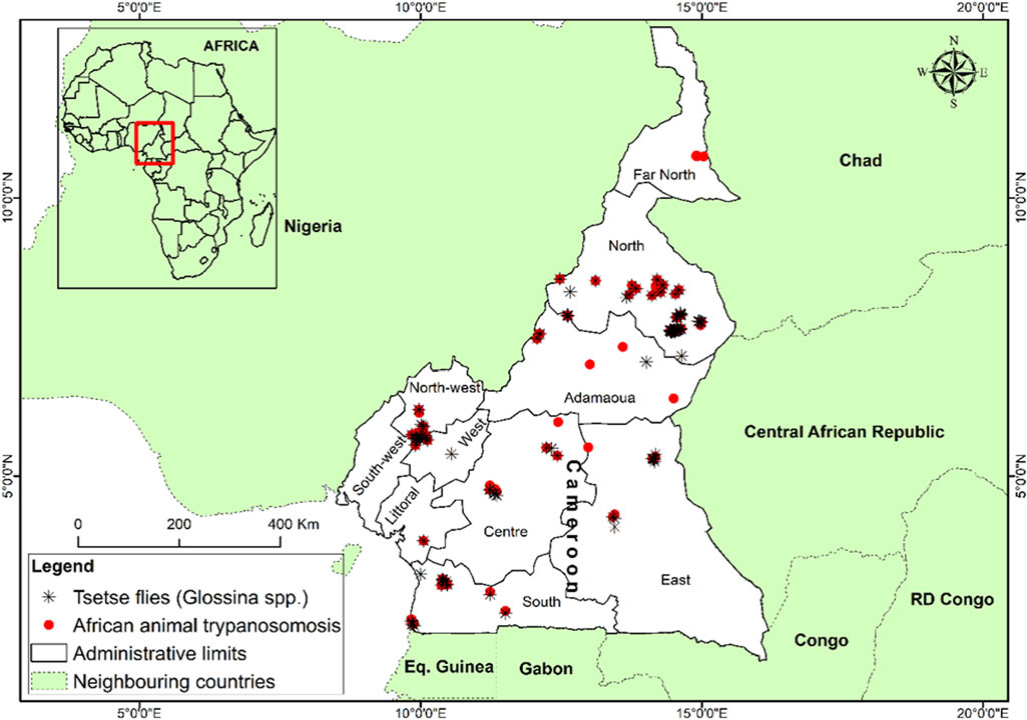
Map showing tsetse flies and AAT distribution in Cameroon.

The map of tsetse flies and AAT of Cameroon indicates that for most regions where tsetse flies are present, animal trypanosomiasis occurs (Fig. 7). It also indicates that in the apparent absence of tsetse flies in the Far North region, AAT still occurs.

## Discussion

AAT is present in the major cattle regions of Cameroon and in the HAT foci, where domesticated and wild animals are considered as reservoir hosts of human pathogenic *T. brucei gambiense* [57, 59, 62]. The majority of documents on tsetse distribution and IR were studies conducted in the northern and southern parts of the country, with >50% published online between 2010 and 2021. Epidemiological data on AAT were not available for the West and Littoral regions and information on tsetse IRs has not yet been documented for Littoral, North West, Far North and East regions. Furthermore, distribution data on tsetse trypanosomal species has not yet been documented for East, Littoral, North West and Far North regions.

For this study, 74 documents presenting relevant information on AAT and tsetse flies from January 1990 to May 2021 were examined, providing a review of datasets involving 19 532 sampled animals of which 70.2% were cattle. The overall AAT prevalence was 21%, indicating that the disease is hyper-endemic in Cameroon. However, the highest prevalence was recorded in pigs, followed by small ruminants, wildlife, cattle and finally dogs. The low prevalence in cattle was not unexpected as in recent decades, AAT control efforts in cattle have been conducted [9]. Similarly, vector control operations are generally directed towards cattle settlement areas [9] because of the socio-cultural, economic and nutritional values of this animal species. Of relevance to the lower trypanosomiasis rate in cattle, it is noted that >90% of cattle in Cameroon are predisposed to infection with AAT, so systematic treatment of cattle has been devised by owners and efforts to free pasture areas from tsetse in cattle settlement zones by the MSEG are on-going. Furthermore, in the Adamawa, North and Far North regions, cattle destined for the transhumance are systematically treated (with trypanocides and sprayed with insecticides) prior to departure and upon return. To the best of our knowledge, trypanosomiasis control operations in Cameroon targeting other animal species have not been conducted.

Regarding the AAT prevalence rates with regions, Adamawa and Centre had the highest rates, with the lowest in the Far North region. The high prevalence for Adamawa was expected as Adamawa is the known epicentre of AAT, with the first case recorded in this region in 1953 [9]. It is also recognised as the point where cattle arriving from neighbouring countries congregate, increasing risks of infection and disease [63]. Adamawa is also recognised as the region supplying the majority of animals and animal products to countries of the Central African subregion and major cities such as Douala and Yaounde [63]. Furthermore, transhumance, where animals are moved in search of fresh pasture areas during the dry season, occurs commonly in the region. As this animal movement enables the mixing of cattle from various regions and countries, and also the movement of cattle between tsetse-free and infested zones [9], there is likely to be increased risks of exposure of cattle to infectious agents, particularly as transhumance routes are mostly adjacent to forest reserves harbouring tsetse fly populations [31], increasing risks of AAT-causing pathogens transmission. Areas in Cameroon where wildlife and livestock population exposure occurs, have recorded high prevalence of AAT, particularly on the Adamawa plateau [34, 64–68]. Tsetse re-infestation of formerly declared tsetse-free zones of the Adamawa plateau by the MSEG is recognised as an on-going issue due to transhumance and trading of cattle [25], accompanied by increased resistance to trypanocides [9, 27, 47] and insecticides [69] used commonly in the region.

The lowest prevalence rate in the Far North region was expected as control of AAT has occurred there since the 1970s, following the successful eradication of tsetse fly in this region [70]. To date, no tsetse fly has been captured in most parts of the Far North region of Cameroon, although biting dipterids including tabanids and *Stomoxys* are abundant [36, 44].

At the AAT species level, *T. congolense*, *T. vivax* and *T. brucei* s.l. were found frequently in the study animals of the various regions and is a similar finding to that of authors from Nigeria, Zimbabwe, Mali and Kenya [71–74]. The epidemiology of AAT in SSA and Cameroon in particular is complex due to several factors. First, there is the co-existence of wild animal reservoir hosts and the presence of biting flies with established vector capacities for mechanical transmission of trypanosomes in the absence of tsetse flies. Second, there has been increasing development of resistance by trypanosome strains to the available trypanocidal molecules. Third, there is the issue of multiple hosts providing a blood meal source for tsetse flies to transmit AAT, with the identification of *T. brucei gambiense* in domestic sheep, goats, dogs, pigs and other species including wild animals (e.g. rodents and primates) having been reported in southern Cameroon [56]. Similarly, *T. congolense* (forest and savannah types), *T. vivax* and *T. brucei* s.l. have been identified in these animals in this part of the country. In addition to serving as hosts for AAT, this broad range of animal species could also serve as reservoir hosts for HAT [75, 76]. Of interest was that the present study identified the limited information on the tsetse blood meal hosts and identification of wild AAT reservoir hosts in the northern part of Cameroon, with only one study identifying AAT in small ruminants, both sheep and goats, in Ngaoundere [77].

The overall ADT of tsetse flies in the nine regions of Cameroon was 1.5 f/t/d. Littoral and South West regions recorded the highest ADT, with the lowest recording in East and West regions. The high abundance of tsetse flies in the Littoral region likely reflects that the survey was conducted adjacent to the Douala-Edea Game Reserve, with forests harbouring abundant tsetse niches, the presence of vertebrate blood meal hosts and an absence of on-going tsetse control operations, of relevance. Similarly, the high ADT for the South West region likely reflects both the presence of both tsetse niches and wildlife blood meal hosts, and an absence of on-going tsetse control operations. The lowest ADT recorded for the East region could reflect that the study sites in this region were in the livestock production and exploitation corporation (SODEPA) ranch where systematic vector control through animal spraying is conducted. The low ADT in the West region could be linked to the limited prospection sites, trap-types and numbers.

The tsetse trypanosomal IR in the six regions of Cameroon was 14.3%. The tsetse flies trapped in the North, Adamawa and Centre regions recorded highest trypanosomal IRs. This was expected as the AAT prevalence rates of these regions were also high. Based on the tsetse flies species distribution, *G. p. palpalis* had a wider distribution range compared to the other species. Riverine species are considered by far to have the broadest geographical distribution, reflecting their ability to adapt to anthropisation and the progressive erosion of natural vegetation [73]. The distribution of savannah species appears to be highly fragmented and confined to a few relatively small protected areas. This distribution pattern for tsetse flies has been observed in other Western countries [71, 78, 79]. Forest tsetse species were also present and could be attributed to the diversity of ecological landscapes of the country, with the presence of gallery forests and forest vegetations in the North and southern regions favouring the breeding of this fly-group. Regarding the distribution within regions of *Trypanosoma* spp. in tsetse flies, three species, *T. congolense*, *T. vivax* and *T. brucei* s.l., were identified in tsetse from West, Centre, South West, South and Adamawa regions. The human pathogenic *T. brucei gambiense* was identified in trapped tsetse flies from southern Cameroon [56].

The molecular identification of *T. grayi*, *T. theileri* and *T. evansi* was achieved for the first time in Cameroon as previous studies were unable to morphologically discriminate them from the well-known *T. congolense*, *T. vivax* and *T. brucei* s.l., partly because the range of primers used in previous studies in the northern and southern parts of Cameroon could not permit their identification. *T. grayi* is a trypanosome of reptiles that could be transmitted mechanically by tsetse flies and other biting flies (tabanids and *Stomoxys*). Although this reptilian trypanosome is reported for the first time in cattle in Cameroon, it has been identified in crocodiles in the southern part of the country [80]. It is suggested that this species could possibly be transmitted from the reptiles in the abundant aquatic and terrestrial ecosystems of Cameroon, to livestock by biting flies and tsetse flies. *T. theileri* is a non-pathogenic trypanosome that has mostly been encountered in ruminants, particularly cattle, sheep and goats. This species has been previously reported in livestock in Cameroon, especially in cattle from Adamawa and North regions [8, 11, 12, 33]. The occurrence of *T. theileri* has also been detected in small ruminants, both goats and sheep, in neighbouring Gabon [81].

Of importance was that *T. evansi* was detected in tabanids captured from tsetse-free Ngaoundere. It is a tissue and blood dwelling parasite that causes the disease ‘surra’, mostly in parasitised camels and other animals, including horses, pigs and occasionally cattle. It is known to be transmitted by sucking flies and the repeated injuries of biting flies, especially tabanids and *Stomoxys*, with tsetse flies serving as mechanical vectors. As *T. evansi* is unable to achieve cyclical development in tsetse flies, transmission is principally mechanical, occurring from the bites of dipterids in endemic areas [11, 12, 19, 20]. In Africa, *T. evansi* is present in tsetse-free areas and in countries where camels are present, including Mauritania, Morocco, Algeria, Tunisia, Libya, Egypt, Sudan, Eritrea and Ethiopia, although is also found in Mali, Burkina Faso, Niger, Nigeria, Chad, Somalia and Kenya [82]. In Sudan, *T. evansi* has been reported to be mechanically transmitted by *Stomoxys* spp. [83]. As camels are known reservoir hosts of *T. evansi* [84], the presence of camels in the tsetse-free area of Ngaoundere could be a source of infestations of *T. evansi* in indigenous cattle and biting insects. The presence of diverse genera and species of biting insects in Ngaoundere of the Adamawa region [51, 84–89] favours mechanical transmission of *T. evansi*.

There was an overlap in the AAT presence and tsetse flies presence data in most regions of Cameroon. This association between these AAT epidemiological parameters demonstrates the previously reported risks of AAT-causing pathogens’ transmission in Cameroon [51]. This study emphasises that tsetse fly and trypanosomiasis occurrence in livestock–wildlife interfaces remains problematic for the expansion of the animal industry in Cameroon and elsewhere in SSA where AAT disease is endemic.

## Conclusions

Pathogenic *T. congolense*, *T. vivax* and *T. brucei* s.l. occurred frequently in animals and tsetse flies. Other trypanosomes were recorded in animals (*T. grayi* in cattle), tsetse flies (*T. theileri* and *T. grayi*) and biting flies (*T. evansi* and *T. theileri* in tabanids) in Cameroon, for the first time. High AAT IRs were recorded in domestic and wild animals. Wild and domestic animals serve as reservoir hosts for the causative agent of Gambian sleeping sickness (*T. b. gambiense*), a parasite that has also been identified as colonising tsetse flies in southern Cameroon. The tsetse fauna of Cameroon comprises nine *Glossina* spp. The riverine *G. p. palpalis* and forest *G. fuscipes fuscipes* had the broadest distribution range with habitat preferences in protected forest and wildlife areas. Savannah tsetse species had a fragmented distribution around cattle settlement areas and wildlife–livestock interfaces. In Ngaoundere of the Adamawa and the Far North region AAT occurs in the apparent absence of tsetse flies. The present study maps indicate that AAT in Cameroon occurs beyond tsetse belts. We recommend that the MSEG should not focus their tsetse interventions on the Far North, North and Adamawa, but should also target other livestock areas of southern Cameroon where AAT and its vectors are present by consulting the maps available in this study.

## Future implications

Future studies will include undertaking a nationwide field entomological and epidemiological baseline data collection to get current information to guide interventions. To conduct a proper socio-economic survey to determine the priorities of livestock farmers and the Government. This data will be very useful for planning and implementing targeted tsetse and AAT control interventions in Cameroon.

## Data Availability

The datasets used and/or analysed during the current study are available from the corresponding author on reasonable request.

## Appendix

**Table A1.** Year and number of documents with complete information on the occurrence of AAT, tsetse and their trypanosomal IRs

Year of publication	Number
1990–1994	2
1995–1999	1
2000–2004	4
2005–2009	11
2010–2014	22
2015–2019	23
2020–2021^a^	11
Total	74

a Up to 15 May.

**Table A2.** Availability of published surveys on, and reported presence of, AAT, tsetse flies and tsetse trypanosomal infection in Cameroon

	AAT		Tsetse flies		Tsetse trypanosomal infection	
Region	Published Surveys	Reported presence	Published Surveys	Reported presence	Published surveys	Reported presence
South	+	+	+	+	+	+
Adamawa	+	+	+	+	+	+
South West	+	+	+	+	+	+
North	+	+	+	+	+	+
Centre	+	+	+	+	+	+
East	+	+	+	+		
Littoral			+	+		
West			+	+	+	+
North West	+	+	+	+		
Far North	+	+				

Published surveys, and the related reported presence, refer to scientific documents. Period of data collection: January 1990–May 2021.
